# Phylogenetic Evidence of Long Distance Dispersal and Transmission of Piscine Reovirus (PRV) between Farmed and Wild Atlantic Salmon

**DOI:** 10.1371/journal.pone.0082202

**Published:** 2013-12-11

**Authors:** Åse Helen Garseth, Torbjørn Ekrem, Eirik Biering

**Affiliations:** 1 Department of Health Surveillance, Norwegian Veterinary Institute, Trondheim, Norway; 2 Department of Natural History, Norwegian University of Science and Technology University Museum, Trondheim, Norway; Auburn University, United States of America

## Abstract

The extent and effect of disease interaction and pathogen exchange between wild and farmed fish populations is an ongoing debate and an area of research that is difficult to explore. The objective of this study was to investigate pathogen transmission between farmed and wild Atlantic salmon (*Salmo salar* L.) populations in Norway by means of molecular epidemiology. Piscine reovirus (PRV) was selected as the model organism as it is widely distributed in both farmed and wild Atlantic salmon in Norway, and because infection not necessarily will lead to mortality through development of disease. A matrix comprised of PRV protein coding sequences S1, S2 and S4 from wild, hatchery-reared and farmed Atlantic salmon in addition to one sea-trout (*Salmo trutta* L.) was examined. Phylogenetic analyses based on maximum likelihood and Bayesian inference indicate long distance transport of PRV and exchange of virus between populations. The results are discussed in the context of Atlantic salmon ecology and the structure of the Norwegian salmon industry. We conclude that the lack of a geographical pattern in the phylogenetic trees is caused by extensive exchange of PRV. In addition, the detailed topography of the trees indicates long distance transportation of PRV. Through its size, structure and infection status, the Atlantic salmon farming industry has the capacity to play a central role in both long distance transportation and transmission of pathogens. Despite extensive migration, wild salmon probably play a minor role as they are fewer in numbers, appear at lower densities and are less likely to be infected. An open question is the relationship between the PRV sequences found in marine fish and those originating from salmon.

## Introduction

Farming of Atlantic salmon (*Salmo salar* L., 1758) is a young, fast-growing and economically important industry in Norway [1] but has not evolved without controversy. Concerns have been expressed by environmental Non-Governmental Organizations, consumers and governmental bodies with regards to animal welfare and health, area-use, pollution, exploitation of marine resources as feed ingredients and the impact of escapees and disease transmission on wild salmonid populations ([2], and references cited therein). During the last four decades when salmon farming has evolved from small scale supplementary enterprises to a multinational industry, the number of returning wild Atlantic salmon has declined [3]. These coincidental events have fed an ongoing debate concerning the potential negative effects of the growing industry on wild salmon populations. Escaped farmed salmon, sea-lice (*Lepeophtheirus salmonis* Krøyer, 1837) infestation and infectious diseases are all regarded as threats to the sustainability of wild salmon [3]. While sea-lice [4]–[6] and escapees [7]–[9] are subject of extensive research, the threat of infectious disease spreading from farmed to wild salmon has received less attention. The introduction and spread of the bacterial disease furunculosis [10], [11] and the monogenean parasite *Gyrodactylus salaris* Malmberg, 1957 in Norway are a few exceptions to this rule [12]–[14].

Evidence of the extent and effect of disease interaction between wild and farmed Atlantic salmon populations has been difficult to obtain. Farmed Atlantic salmon have their origin in wild populations and, needless to say, so do most of the pathogens that cause diseases in farmed fish [15]. However, in contrast to the farm environment, conditions that promote epidemics and disease outbreaks, such as high host density, are rarely found in wild populations. As a consequence, farmed salmon are likely to account for higher levels of pathogen production, transmission and virulence evolution than wild salmon [15]–[18]. It is difficult to study the effect of pathogen transmission from farmed to wild salmon, partly due to methodological challenges as infected wild fish often die and disappear before they are detected [18], [19]. The versatile life cycle of wild salmon also implies that they are affected by multiple factors, other than infectious diseases, that can cause populations to decline. These factors may act locally such as acidification [6], or at a larger scale such as climatic change [20], [21] and availability of food in the ocean [20]. The outcome of these factors may camouflage potential adverse effects caused by pathogen spill from farmed salmon as they are all registered as reduced marine survival.

Molecular epidemiology has been used to investigate the dissemination and evolution of human viruses [22], [23], conduct epidemiological research within the aquaculture industry [24]–[27], and is proposed as a tool useful for investigations of wild-farmed disease interaction [28]–[31]. The objective of this study was thus to investigate pathogen transmission between farmed and wild Atlantic salmon populations in Norway by means of molecular epidemiology. Piscine reovirus (PRV) was selected as the model organism as it is widely distributed in both farmed and wild Atlantic salmon in Norway. PRV is also a suitable model as infection not necessarily will lead to loss of study subjects through development of disease and mortality [32].

PRV is a reovirus associated with the development of heart and skeletal muscle inflammation (HSMI), a common and commercially important disease in farmed Atlantic salmon in Norway [33]–[35]. HSMI has also been found in farmed salmon in Scotland [36]. PRV is detected in both healthy and diseased salmon and appears to be ubiquitous among farmed Atlantic salmon [37]. However, the tissue distribution and increasing viral loads during an HSMI outbreak strongly support a causal relationship between PRV infection and development of HSMI [35], [37]–[39]. Outbreaks of HSMI have so far not been associated with particular strains of PRV [37], and HSMI has not been recorded in wild Atlantic salmon, although PRV seems to be widely distributed in Atlantic salmon and to a lesser extent in sea-trout in Norwegian rivers [32], [40].

Most viral agents that cause disease in salmonids in Norway have genomes consisting of RNA. Due to a higher mutation rate than DNA, the virus genome can change considerably over a relatively short period of time. This results in a high RNA-virus variability that can be used as a tool to trace spread of viral infection by the use of molecular epidemiology [41].

Reoviruses are icosahedral and non-enveloped with double-stranded RNA genomes of 10–12 segments. The *Reoviridae* consists of two subfamilies, *Spinareovirinae* and *Sedovirinae*, with altogether fifteen genera [42]. The host range of *Reoviridae* extends from insects, plants and fungi to fish, molluscs, reptiles, mammals and birds. Piscine reovirus was originally described as equally distant to genera *Orthoreovirus* and *Aquareovirus* in the subfamily *Spinareovirinae* [35]. PRV has 10 gene segments similar to *Orthoreovirus* [43], and two recent studies suggest that PRV is more closely related to *Orthoreovirus* than to *Aquareovirus* [44], [45]. Hence the name *Piscine orthoreovirus* has been suggested [45]. However, a recent whole genome analysis concluded that PRV should be considered as member of a new genus within the family *Reoviridae* [46]. The same study also reports that PRV segment S1 sequences group into one genotype with two separate sub-genotypes, both found in Norway [46]. Recent research indicate that S1 is bicistronic encoding σ3 (a 330 amino acid (aa) outer capsid protein), and p13 (a 124 aa cytotoxic, nonfusogenic integral membrane protein) [44], [45]. S2 is also possibly bicistronic encoding the 420 aa inner capsid protein σ2 and p8 (a 71 aa hypothetical protein) [35], [45]. S4 is monocistronic encoding σ1 (a 315 aa cell attachment protein) [35], [44], [45].

In this study molecular epidemiology is used to investigate transmission of PRV between farmed and wild Atlantic salmon populations in Norway. Pathogen exchange can occur between Atlantic salmon stocks during marine migration, due to wild fish straying from neighboring rivers or by escapes from aquaculture. Finally, the presence of PRV in sea-trout [32] and marine species [47] raise questions regarding their role in pathogen exchange with Atlantic salmon.

## Materials and Methods

### Ethics statement

Samples utilized in this study and the preceding cross sectional survey of piscine reovirus infection [32] are residuals of samples originally intended for infectious pancreatic necrosis virus (IPNV) testing of brood fish as part of statutory health control in stock enhancement hatcheries and the Norwegian gene bank for wild Atlantic salmon. Additional residual samples were obtained from infectious salmon anaemia virus (ISAV) and viral hemorrhagic septicaemia virus (VHSV) surveillance conducted in wild salmonid populations. Hence, these samples represent secondary use of available material from existing health monitoring activities.

Samples from four escaped farmed salmon from river Etne (2010) were obtained during organised recapture after an escape from a nearby aquaculture site. The County Governor of Hordaland gave permission to the recapture (Fiskeløyve 23-2010).

All fish were killed in accordance with the Norwegian animal welfare act. Brood fish were anesthetized with trikainmesilat (metacaine) or benzocaine and killed by exsanguination. All other animals were stunned by a blow to their head and killed by exsanguination. No animals were killed specifically for this study. The authors have permission to use all samples.

### Study sample and selection criteria

The majority of samples were from a cross-sectional survey of piscine reovirus infection described by Garseth and co-workers [32]. The survey was based on quantitative RT-PCR screening of head kidney samples from 1207 returning spawners of Atlantic salmon and 133 sea-trout captured in 36 rivers from 2007 to 2009. A total of 200 Atlantic salmon and four sea-trout (*Salmo trutta* L.) were PRV-positive. In addition, four escaped farmed salmon from river Etne (2010) were included in the study. These were caught during organised recapture after an escape from a nearby aquaculture site and are thus believed to originate from this site.

Scale-circuli patterns [48]–[50] and knowledge of local cultivation and release practices were used to determine the origin (*life-history*) of the Atlantic salmon. Hence, the term *wild* describes individuals that are the result of natural spawning and recruitement in the river, the term *escaped farmed* describes individuals displaying scale-circuli patterns of salmon escaped from commercial aquaculture, while the term *hatchery-reared* describes individuals that are offspring of wild parents but reared in hatcheries and released for stock enhancement or restoration purposes [32].

The selection criteria were chosen to agree with the objective of the study; to investigate pathogen transmission between wild and farmed salmon populations. Hence, salmon from all counties and life-histories were included. In addition, only samples from PRV-positive salmon with cycle threshold (C_t_) values below 30 were included to ensure good sequence quality. However, all four PRV-positive sea-trout (C_t_ -values 25.9–39.5) were included in the initial amplification step. Sequences generated in this study are deposited in the European Nucleotide Archive with accession numbers HG329842 to HG330021 (http://www.ebi.ac.uk/ena/data/view/HG329842-HG330021).

All tissue-samples and RNA-extracts used in this study are deposited in the collections of the Norwegian University of Science and Technology (NTNU) University Museum and at the Norwegian Veterinary Institute, section for environmental and biosecurity measures.

### RNA-extraction, RT-PCR amplification and sequencing

RNA was extracted from head kidney tissue as described by Garseth and co-workers [32]. RNA was isolated from approximately 20 mg of tissue with MagMAX TM-96 Total RNA Isolation Kit (cat #1830, Ambion). The subsequent RNA extraction was performed according to the manufacturers' recommendations with the same kit. A KingFisher (Labsystems Oy) was used in the magnetic-based separation. After elution, RNA concentration and purity was measured by use of NanoDrop ND-1000 spectrophotometer (NanoDrop Technologies). All samples had OD260/280 ratios between 1.97 and 2.12 (mean 2.06). Four aliqots à 15 µl eluated RNA were produced from each sample, one of these were used in the initial qRT-PCR PRV screening and three were frozen at −70°C. Altogether 91 samples were selected for amplification and transferred to NTNU University Museum on dry ice. Piscine reovirus genome segments S1, S2 and S4 were selected for amplification and sequencing (based on recommendations from Espen Rimstad and Torstein Tengs, coauthors of [35]).

An overview of analysed gene segments, primer combinations and primer sequences is shown in Table 1.

**Table 1:** Primers and their combination used in amplification and sequencing of segments S1, S2 and S4 of the piscine reovirus genome.

Genome segment	Primer set	Forward primer	Primer sequence	Reverse primer	Primer sequence
S1	S1 No 1	S1_39F	AAACCCAAATGGCGAACCA	S1_621R	TGCTCCACTGGGTTCAGCTC
	S1 No 2	S1_460F	TTGAAGCTAAGCGACGCCTT	S1_1036R	ACAGTAGGCTCCCCATCACG
	S1 No 3	S1_39F	AAACCCAAATGGCGAACCA	S1_1036R	ACAGTAGGCTCCCCATCACG
					
S2	S2 No 1	S2_43F	TGGCTAGAGCAATTTTCTCGG	S2_720R	GCCATTCCATGTCATCGTTG
	S2 No 2	S2_603F	TCGGTGCACGATATGAAAGC	S2_1304R	GTGGTCAGTCCCGGCTAGAG
	S2 No 3	S2_43F	TGGCTAGAGCAATTTTCTCGG	S2_1304R	GTGGTCAGTCCCGGCTAGAG
					
S4	S4 No1	S4_30F	TTAACCGCAGCGACATCTCA	S4_591R	TTGGTGCCGTCCCAACA
	S4 No 2	S4_456F	ACTGACCTGCTTGGACACACTG	S4_1005R	GACACGTGGCTCTTCCACG
	S4 No 3	S4_30F	TTAACCGCAGCGACATCTCA	S4_1005R	GACACGTGGCTCTTCCACG

Reverse transcription and PCR amplification of S1, S2 and S4 were carried out in one step with QIAGEN OneStep RT-PCR kit (QIAGEN AB) using the primer combinations in Table 1. S1 was initially amplified using primer set 3 enabeling a near full length amplification. This approach was abandoned as sequence quality was improved by the amplification of S1 in two overlapping fragments (using primer sets 1 and 2). 2 µl template (2–10 ng total RNA), 1.5 µl forward primer and 1.5 µl reverse primer (final concentration 4 pmol/µl) was denaturated for 5 min at 95°C before 19.85 µl primer free Mastermix (QIAGEN OneStep RT-PCR kit) and 0.15 µl RNAse Out (Invitrogen) were added. The following PCR conditions were used: 30 min at 50°C (reverse transcription): 15 min at 95°C (inactivation of reverse transcriptase and activation of hot-start PCR DNA polymerase): 30 sec at 94°C (template denaturation): 30 sec at 55°C (primer annealing): 1 min at 72°C (fragment elongation). Steps 3–5 were repeted 40 times followed by a final elongation step of 3 min at 72°C.

Gel electrophoresis in 1% agarose gel with SYBR Safe stain (Invitrogen) was used to test the success of the amplification and served as an additional criterion for selecting samples for sequencing.

PCR products selected for sequencing were purified with ExoSAP-IT (USB Products) to remove excess nucleotides and unincorporated primers. Selected samples were sequenced bi-directionally by cycle sequencing technology using dideoxy chain termination/cycle sequencing on ABI 3730XL sequencing machines at Eurofins.

Amplification and sequencing was conducted twice for a proportion of the samples as a test of lab routine quality. For S1, 8 sequences were run twice, 15 were run twice for S2 and 19 were run twice for S4. Altogether 42 sequences were run twice, and of these 40 were identical while 2 had too low quality in the second run to be compared with the sequences from the first run. In total, 27 of the 180 sequences (15%) selected for the final dataset were included in this quality control.

### Sequence editing and alignment

DNA sequences were assembled and edited with DNABaser Sequence Assembler v3.5.0 2011 (Heracle BioSoft SRL, http://www.DnaBaser.com). Sequences were assembled automatically and inspected and edited manually. In cases of ambiguity of base calls, the appropriate International Union of Pure and Applied Chemistry (IUPAC) code was inserted. Edited nucleotide contigs were imported to MEGA5 [51] and aligned as codons by MUSCLE [52] under default settings. Alignment was trivial since no internal indels were observed. Both ends of the alignments were trimmed to remove primers and parts with low sequence quality and indistinct base calls. Translation of nucleotides to amino acid sequences gave complete coding sequences.

For all three segments a standard nucleotide NCBI BLAST search (blastn) was conducted to identify and add available sequences of aquaculture origin to the alignments. Altogether 10 sequences were obtained from GenBank, whereof three were consensus sequences deposited in GenBank by Palacios and co-workers [35]. These were not included in the alignments as geographic origin was a key selection criterion. The remaining seven sequences (accessions JN991006-JN991012) were PRV S1 sequences derived from an industry based study conducted in Norway [37]. Information with regards to geographic origin of these samples was obtained from the authors and anonymized by limiting information to county of origin.

### Phylogenetic analysis

Description of marker composition and initial maximum likelihood phylogenetic analyses were made on all three genomic segments in MEGA5 using 1000 bootstrap replicates and the Kimura 2-Parameter model (K2) on S1 and S2 [53] and K2 with Gamma correction on segment S4. However, rigorous phylogenetic analyses were performed on S1 and the concatenated dataset only, as these were the alignments containing sequences derived from farmed Atlantic salmon. Moreover, S1 was the most phylogenetically informative segment in our dataset (Table 2).

**Table 2:** Basic statistics on the reverse transcribed genome segments used in the phylogenetic analyses of Norwegian piscine reovirus strains.

Genome segment		S1	S2	S4	Total
Nucleotides	Length of segment (bp)	1081	1329	1040	3450
	Length of segment used in analyses (bp)	837	1182	879	2898
	Conserved sites (bp)	785	1140	843	2768
	Variable sites (bp)	52	42	36	130
	Parsimony informative sites (bp)	43	28	28	99
	A (%)	27.4	23.8	25.4	25.4
	C (%)	23.9	24.0	23.2	23.7
	G (%)	25.7.	24.5	26.1	25.3
	T (%)	23.0	27.7	25.3	25.5

The best fit substitution model and partition scheme was found using PartitionFinder 1.0.1 [54] testing for all substitution models and all possible combinations of markers and nucleotide positions. The best partition scheme according to the Bayesian Information Criterion (BIC) on the concatenated dataset contained three partitions, consisting of nucleotides from 1st position, 2nd position and 3rd position for all markers. The best substitution models on these partitions were the Hasegawa, Kishino and Yano model [55] with a proportion of invariable sites (HKY+I) on partition 1 and 2, and HKY+G+I (including gamma corrections for rate variation among sites) on partition 3. The best partition scheme for the marker S1 alone contained two partitions: 3rd position and 1st + 2nd position, both with the Kimura 2-Parameter model [53] as the best fit substitution model. Maximum likelihood (ML) analyses with 1000 bootstrap replicates were run on the partitioned dataset in RAxML 7.4.2 [56] utilizing the software raxmlGUI [57]. Since the best fit substitution models are not implemented in RAxML, we used the GTR+G model in our analyses.

Phylogenetic analyses by Bayesian inference were performed on the partitioned datasets in MrBayes 3.2.1 [58], [59]. The Metropolis-Coupled Monte Carlo Markov Chain method with default four independent chains (nchains = 4) was run for 3,000,000 generations (ngen = 3,000,000). The frequency with which the chains were swapped was set to 0.2 (temp = 0.2). Every 200 generations a tree and corresponding parameter values were sampled and recorded to file (samplefreq = 200). The first 25% of sampled trees were discarded as the burnin fraction (relburnin =  yes burninfrac = 0.25). Effective sample size (ESS) estimated with Tracer v1.5.0 [60] and standard deviation of split frequencies (≤0.01) were used as convergence diagnostic. For S1 and the concatenated dataset 50% majority rule consensus trees (contype = halfcompat) were constructed from the tree output files. Phylogenetic analyses on reduced datasets that only contained information from synonymous sites were also conducted. These were run with the same setup as the full datasets described above but without partitioning. The estimated phylogenetic trees were visualised in Figtree v1.3.1 [61] and MEGA5.

## Results

### Sequence composition and description of alignment

For all three genomic segments the final alignment matrix comprised sequences from 27 rivers with wild (N = 45) and hatchery-reared (N = 6) Atlantic salmon, one anadromous trout (sea-trout) and eight escaped farmed salmon whereof four were captured in river Etne in 2010 during an escape from a nearby aquaculture site. In addition, S1 and the concatenated alignment also comprised the seven sequences from GenBank derived from six cohorts of farmed Atlantic salmon from five counties. The final matrix of aligned sequences is described in Tables 2 and 3.

**Table 3:** Overview of origin of samples used in phylogenetic analyses.

Sample category		Samples	PRV-positive	Phylogeny	Rivers/sites represented
Sea-trout		133	4	1	1
Atlantic salmon	Wild	1008	134	45	24
	Hatchery reared	124	30	6	2
	Escaped farmed	61	33	4	4
	Uncertain	14	3	-	-
	Escaped farmed Etne*	38	37	4	1
GenBank	Farmed	-	-	7	6 (5 counties)

The final alignment comprised PRV protein coding sequences S1, S2 and S4 from sea-trout, wild, hatchery-reared and farmed Atlantic salmon.

* From Etne; believed to come from the same aquaculture site.

None of the three nucleotide alignments (S1, S2 and S4) contained insertions or deletions. As described in Table 2, 837 of 1081 (77.4%) nucleotides were used in the analyses of PRV S1. This genome segment was the most variable segment with 52 (6.2%) variable sites whereof 43 (5.1%) were parsimony informative. For S2 1182 of 1329 (88.9%) nucleotides and for S4 879 of 1040 (84.5%) nucleotides were used in the analyses.

### Phylogenetic analysis

**Figure 1:**
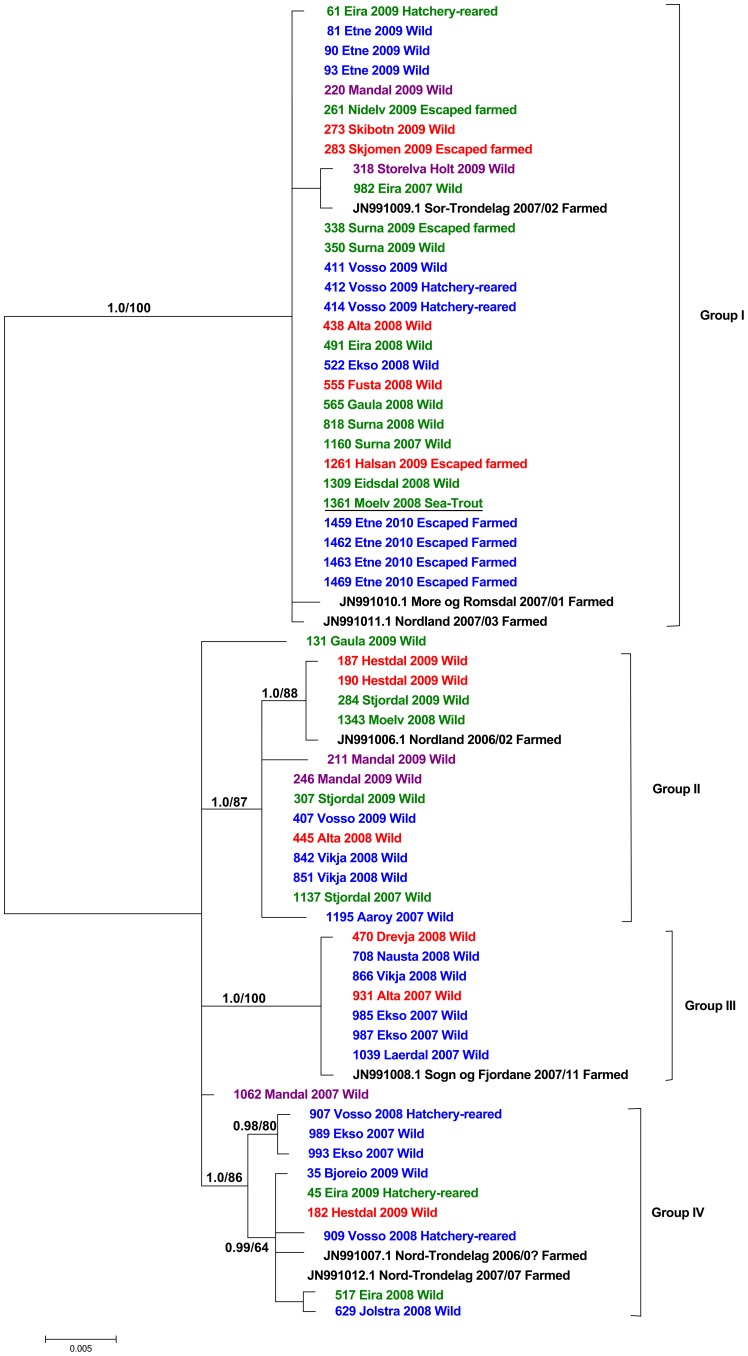
Resulting phylogenetic tree derived from Bayesian analysis of protein-coding PRV genome segment S1. Numbers above branches refer to Bayesian posterior probabilities and bootstrap support from corresponding maximum likelihood, respectively. Samples are identified with ID-numbers, geographical origin, year of sampling and life-history. Colours are corresponding to geographical regions in Figure 3. Sequences representing farmed Atlantic salmon are in black and marked with their respective GenBank accessions, county of origin and life history. Sequence from *Salmo trutta* is underlined.

The initial phylogenetic analysis of S1 returned a result nearly identical to the tree in Figure 1.

**Figure 2:**
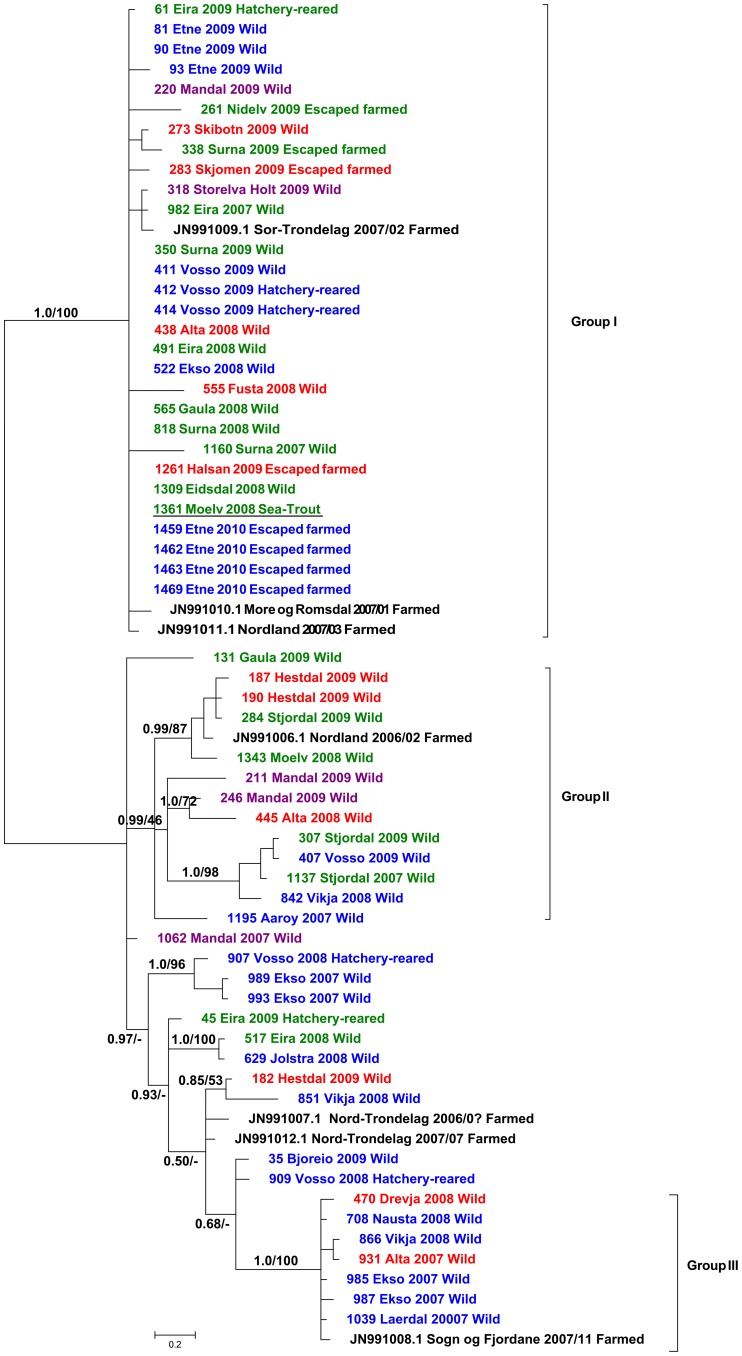
Resulting phylogenetic tree derived from Bayesian analysis of the concatenated dataset. The dataset contained protein-coding PRV genome segments S1, S2 and S4. Numbers above branches refer to Bayesian posterior probabilities and bootstrap support from corresponding maximum likelihood analysis, respectively. Samples are identified with ID-numbers, geographical origin, year of sampling and life-history. Colours are corresponding to geographical regions in Figure 3. Sequences representing farmed Atlantic salmon are in black and marked with their respective GenBank accessions, county of origin and life history. Sequence from *Salmo trutta* is underlined.

The result from rigorous Bayesian and ML analyses of S1 and the concatenated dataset are concordant and support the same groups (Figures 1 & 2). For the concatenated dataset, three major groups and several minor clades are well supported, with the exception of Group II in ML analyses (Figure 2). The same major groups are evident in the result based on analysis of the S1 dataset, but here an additional Group IV is also well supported. The initial analyses of segments S2 and S4 presented largely non-conflicting patterns to S1, but groups as defined in the phylogenetic analysis of S1 are not recovered in the same degree. Generally branches have lower support and are shorter (Figure S1 & Figure S2).

**Figure 3:**
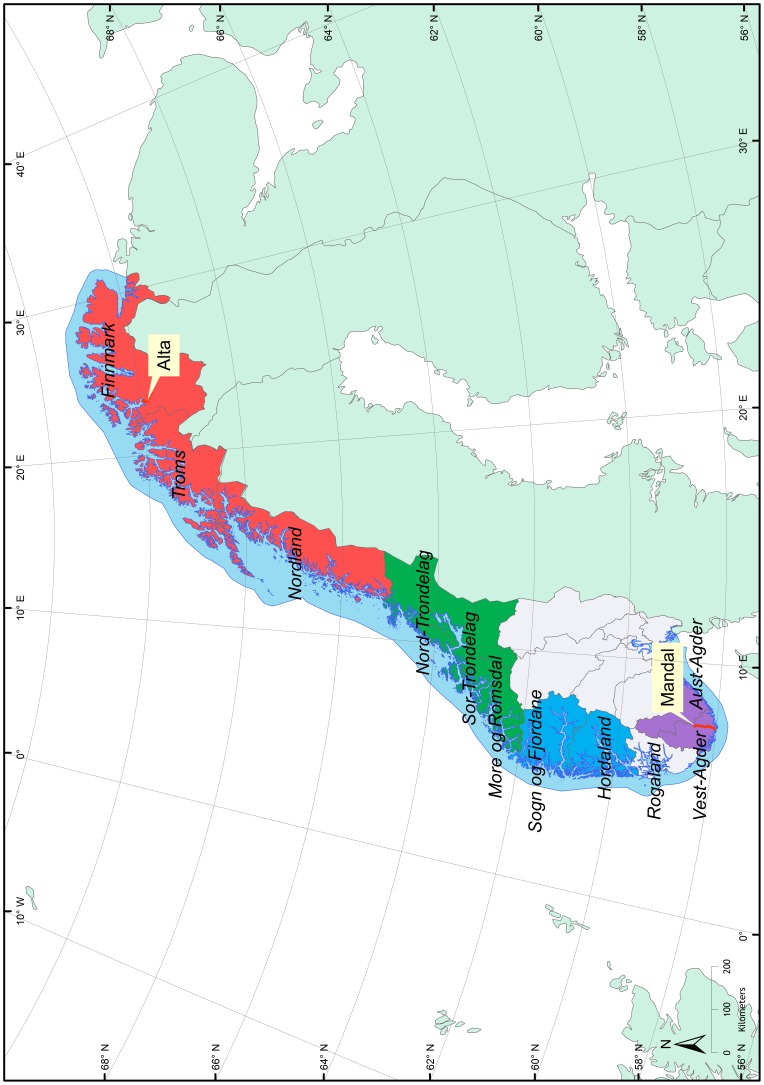
Map of Norway showing rivers Alta and Mandal and counties. Counties are color coded according to geographic region.

With the exception of a few smaller groupings, for instance the Vosso-Ekso clade in Group IV, there is very poor geographical structuring in our trees. All 3-4 main groups include samples from wild stocks (i.e. rivers) situated geographically far apart. For instance, wild salmon from rivers Alta (69°N, red) and Mandal (58°N, purple) appear together in Group I and II even if they are situated 1800 km apart (see Figure 3 for geographic location). The rivers Storelva Holt (purple southern region) and Skibotn (red northern region) are both present in Group I, and river Hestdal (red northern region) appear together with samples from river Vosso (blue western region) and Bjoreio (blue western region) in Groups II and IV respectively (Figures 1 & 2).

Further investigation revealed that S1 sequences were identical in multiple salmon from the same rivers and that in a subset of these S1, S2 and S4 were identical. In two of these rivers, sequences were obtained from salmon that according to the local stock enhancement hatcheries were cohabitants in the same tank before stripping and sampling. In a third river, identical sequences came from a hatchery where salmon were moved between several tanks. Hence, the identical sequences could be caused by infection during cohabitation.

The PRV S1 sequences from farmed Atlantic salmon, representing six aquaculture cohorts from five different counties, are dispersed among all four main groups along with sequences obtained from wild, hatchery-reared and escaped farmed salmon (Figures 1 & 2). In Group I, three sequences from farmed salmon representing three aquaculture cohorts from three counties appear together (Figures 1 & 2). In Group IV, two sequences from farmed salmon representing the same aquaculture cohort (fresh water and sea-water phase) group together (Figure 1). Groups II and III each have one sequence originating from farmed Atlantic salmon. PRV obtained from escaped farmed salmon captured in river Etne in 2010 group together in Group I indicating that at the point of escape there was limited within-site variation. Finally, PRV from sea-trout group together with PRV from Atlantic salmon in Group I (1361 Moelv 2008 Sea-trout).

Phylogenetic analyses of the synonymous sites from the S1 and concatenated datasets resulted in trees with the same general pattern (Figure S3 & Figure S4).

## Discussion

### Phylogenetic evidence of pathogen transmission between populations

Due to the assumed functions of the genome segments analysed, it is likely that their protein products are subject to natural selection, especially from the hosts' immune systems. This violates the assumption of neutral markers in phylogenetic reconstructions and could potentially strongly influence the relationship between virus strains. To investigate if variation in non-synonymous sites influenced our results, we ran the same analyses on reduced datasets only incorporating synonymous sites. Although some resolution was lost and some groups received slightly lower support values (Figure S3 & Figure S4), the results of these analyses were concordant with the results from the full datasets. We therefore conclude that the relationships seen between PRV strains in our data is not significantly influenced by converging or parallel evolution.

Geography was a key criterion in planning and conducting the study. This was based on the hypothesis that if distinct host populations are isolated geographically and there is no pathogen exchange between them; pathogen sequences will group according to the geographic origin of the host. Our results strongly indicate pathogen exchange between distant populations of Atlantic salmon, as PRV sequences from these populations are placed together in well supported genetic clusters.

Pathogen exchange can occur between Atlantic salmon stocks during marine migration, and be caused by straying from other rivers or escapes from aquaculture. Finally, the presence of PRV in sea-trout and marine species raise the question regarding their role in pathogen exchange with Atlantic salmon.

### Pathogen exchange between wild Atlantic salmon stocks

Most Atlantic salmon spawners return to the river they left as smolts. This has led to genetically distinct salmon stocks or even several distinct populations in each river [62]. Contact between wild salmon stocks can occur during migration, straying and within live gene banks for wild Atlantic salmon (see below).

The marine feeding migration is the least studied phase of Atlantic salmon life, and information regarding the spatial and temporal distribution during this period is scarce. Dadswell and co-workers [63] reviewed data accumulated during the last five decades and concluded that the most probable marine migration model is the “Merry-Go-Round”- hypothesis. This hypothesis proposes that North-American and European stocks enter the North Atlantic Sub-polar Gyre from their respective sides of the Atlantic and migrate counter clockwise until they return to their native river [63]. It is difficult to estimate the extent of interaction and the potential for pathogen transmission between individual salmon and stocks during migration. Catch rates from Faroese long-line fisheries from November 1981 to May 1982 showed that from 0 to 286 salmon were caught per 1000 hooks, and during the west Greenland fisheries (before 1980) the highest catches were 20–70 salmon km^−1^. This indicates that salmon occur in small shoals and provides some insight about density. However, these data were generated before the major decline of wild populations had occurred. Scale discrimination and marking studies indicate a mixed stock structure, i.e. representation from both European and North-American stocks among catches throughout the North Atlantic Sub-polar Gyre. Tagged adult salmon from the Faroes were recovered in Canadian rivers while marked North American smolt were captured off the Faroes, west and east Greenland and Norway [63]. This indicates that pathogen exchange may occur between individuals and stocks of diverse origin. Still, extensive transmission during migration seems unlikely when the low host density in wild salmon populations is taken into account.

About 3–6% of wild salmon and 15% of hatchery-reared salmon may stray to other rivers during homeward spawning migration [64], [65]. Studies show that most of them enter nearby rivers. For instance, 96% of straying Imsa salmon entered streams within 420 km, and 80% entered streams within 60 km of the mouth of the River Imsa [65]. Hence, pathogen exchange between wild stocks from nearby rivers can happen due to straying.

Norway has established three gene bank stations for live Atlantic salmon to facilitate conservation and restoration of endangered wild stocks. Each gene bank harbour several stocks mainly of regional origin, and a biosecurity strategy has been implemented to minimize the risk of horizontal and vertical pathogen transmission. Founder stocks are established and maintained in the gene bank by importing disinfected, fertilized eggs from wild brood fish that have been subject to pathogen testing and health control. Stocks from different rivers are kept in separate tanks throughout the lifespan, and only disinfected, fertilized eggs from these are exported back to the river. Since stocks within each gene bank are of regional origin, the phylogenetic pattern caused by pathogen transmission within the gene banks cannot be distinguished from the pattern caused by straying. A phylogenetic pattern derived from pathogen dissemination through straying or gene banking cannot be excluded in any of the groups (Figure 2). Still, the pattern is systematically violated by sequences from farmed and wild salmon from other geographic regions.

### Pathogen exchange between wild and farmed Atlantic salmon

Grouping of PRV S1 sequences from farmed Atlantic salmon together with sequences obtained from wild, hatchery reared and escaped farmed salmon indicates that wild and farmed salmon harbour the same virus strains and that virus have been exchanged between populations of different origin. Pathogen exchange between farmed and wild Atlantic salmon can occur by interaction between wild and *escaped* farmed salmon during marine migration or spawning, but also when wild salmon pass through areas with aquaculture production during sea-ward migration as post-smolt or homeward migration as spawners.

Simulated escapes of farmed Atlantic salmon show that migratory behavior of escaped farmed salmon depends on the development stage at point of release. Post-smolt released during early summer migrates out of the fjord whereas post-smolt escaping during late summer and autumn were recaptured in the fjord [66]. Escaped large salmon have the capacity for long distance migration and are recaptured in rivers [67].

Wild and escaped salmon stay in the same parts of the river and interbreeding is known to occur [7], [68]. In addition, congregation of salmon under natural or man-made migration barriers can facilitate pathogen exchange as demonstrated by furunculosis induced mass mortality in Norwegian rivers [11].

Investigation of transmission of HSMI, conducted prior to the description of PRV, confirm horizontal transmission as an important route [69]. Horizontal transmission between sites can occur through virus dispersal by ocean currents, sharing of personnel and equipment, but also through wild fish movements if these are susceptible to the pathogen in question [70].

PRV is ubiquitous in salmon farms [37] and higher odds of PRV-infection in escaped farmed salmon than wild salmon (odds ratio 7.3 p<0,001) is considered both plausible and expected based on the number of HSMI-outbreaks and the PRV-prevalence in farmed fish [71]. Since reoviruses are hydrophilic, non-enveloped viruses and considered relatively robust outside the host [72], [73] it is plausible to suggest that PRV can be abundant in sea-water near aquaculture sites. This makes it possible for wild salmonids to contract PRV-infection as they pass aquaculture sites. Research with salmonids carrying acoustic tags show that while sea-trout stay near aquaculture sites and move between them, wild Atlantic salmon post-smolt and Atlantic salmon x sea-trout hybrids pass the same sites without delay or inter-site movement [74].

The fact that PRV-sequences from wild Atlantic salmon in rivers Mandal and Alta appear together in Groups I and II (Figures 1 & 2), could be explained by transportation of PRV-carrier fish within the Atlantic salmon industry. The structure of the Norwegian salmon farming industry is to a high degree dependent upon transportation of live fish. Fertilized eggs are moved from broodfish stations to hatcheries. Smolt are moved from smolt production sites to on-growing sites in sea-water, and full-grown salmon are transported to the abattoirs for slaughtering. Some of these movements represent long distance transportation. A public record of live fish movement has not been established in Norway; hence detailed information is not available. However, the discrepancy between smolt production and input to sea in most counties is an indication of trans-county movement [75]. Likewise is the discrepancy between production and slaughter capacity in the different counties. The production of smolt in the northern part of Norway (counties Nordland, Troms and Finnmark) has so far not been able to meet the demand of the local industry. This imbalance has been solved by moving smolt from the southern part of Norway. Cases of disease outbreaks have been known to occur after such fish movements; the first cases of pancreas disease in the northern part of Norway occurred in smolt imported from the endemic area in south-western Norway [76], [77].

Farmed salmon far outnumber wild salmon in Norway. At the end of 2011, a total of 366 million individuals (679 398 metric tons) farmed Atlantic salmon were in cages along the coast of Norway [75]. The same year 500 000 wild salmon returned to Norwegian rivers [78], whereof 45% were captured and killed in rivers and fjords. In the rivers escapees constituted approximately 4% of salmon caught during angling season in 2011 (compared to 6–9% the previous 10 years). During the spawning season later that year 12% were escapees (compared to 11–18% the previous 13 years) [78].

The Norwegian salmon farming industry experiences large disease related losses [79] and a considerable proportion of sites experience disease outbreaks with potential pathogen spill over to the environment. In 2011 there were 440 recorded disease outbreaks caused by viral agents [79] in approximately 1000 licensed sites for grow-out production of Atlantic salmon [75].

Fish-farming constitutes a favorable environment for within- and between sites transmission of pathogens. Fish are held at high stocking densities in open cages during the sea-water phase, and sites are connected to nearby sites by coastal currents, movement of fish and sharing of equipment and personnel. The near endless access to susceptible hosts in high densities in the farm environment will keep infections alive over an extended period. This will not only increase the likelihood of pathogen spill over to the environment, but also increase the potential for evolution of more virulent strains [17]. Thus, farmed salmon seem to outnumber wild salmon not only in sheer numbers, but also in the potential for propagation and spread of infectious agents. While diseased farmed salmon are fed and protected against predators in cages, diseased wild fish will strive to catch their prey and to avoid predators. Hence by implication, diseased wild fish will to a greater extent succumb to infections.

### The role of sea-trout

Between 1.9 and 3.0% of sea-trout are PRV-infected [32], [40], and PRV obtained from one sea-trout group together with PRV obtained from Atlantic salmon (Group I). Although only one sequence was available for phylogenetic analyses, this may indicate that sea-trout can play a role in pathogen exchange with and between Atlantic salmon. Although some individuals migrate out of the fjords, most sea-trout spend their entire marine phase in the fjords. Research conducted by the Norwegian Institute for Water Research (NIVA) show that they stay temporarily around aquaculture sites, but also connect different sites by moving between them [74]. Sea-trout are also in close contact with wild Atlantic salmon in the rivers. As most sea-trout limit their marine migration to the fjord, and few of them are infected, sea-trout will *per se* not contribute to the lack of geographic pattern in the phylogenetic tree. However, they can be a link between farmed salmon, which may have been transported long distances, and the local wild salmon stock. Further sequencing, phylogenetic analyses and research are needed to conclude on the role of sea-trout.

### Pathogen exchange between marine fish species and wild Atlantic salmon

Wiik-Nielsen and co-workers [47] screened a total of 1627 fish (379 pools) from 37 different wild marine species using a PRV-specific RT-qPCR assay. Pools from four species yielded positive results; *Argentina silus* (Atlantic Argentine, 1 of 38 pools), *Trachurus trachurus* (Atlantic horse mackerel, 1 of 1 pools), *Mallotus villosus* (Capelin, 1 of 16 pools) and *Clupea harengus* (Atlantic herring, 1 of 37 pools). The highest viral load was detected in herring. To this point virus from marine species have not been sequenced, and it is not known if they represent a marine genotype. Caplin and 0+ herring are important prey for Atlantic salmon post-smolt [80], while 1+ herring often compete with post-smolt for food and occur in high densities in the same habitat in both fjords and the open ocean. Atlantic salmon are hence caught as by-catch in herring surface trawls [81]. Accordingly, the possibility of pathogen exchange between these marine species and Atlantic salmon cannot be excluded and will be better understood when PRV-sequences from marine fish are available.

## Conclusion

This study pinpoints the complex nature of research concerning pathogen exchange between farmed and wild Atlantic salmon. Many factors influence the life and survival of wild salmon and should be accounted for before a conclusion is drawn.

In the present study, PRV serves as a model of pathogen exchange between different wild and farmed populations of Atlantic salmon. PRV is a suitable model organism because it is widely distributed in both populations and because it doesn’t necessarily lead to loss of study subjects through development of disease and mortality. We conclude that the lack of a geographical pattern in the phylogenetic trees is caused by extensive exchange of PRV. In addition, the detailed topography of the trees indicates long distance transportation of PRV.

Through its size, structure and infection status, the Atlantic salmon farming industry has the capacity to play a central role in both long distance transportation and transmission of pathogens. Despite extensive migration, wild salmon probably play a minor role as they are fewer in numbers, appear at lower densities and are less likely to be infected. An open question is the relationship between PRV-sequences found in marine fish and those from salmon.

In this study we have used PRV as a model for pathogen dissemination, and the study strongly supports the existence of pathways for pathogen transmission between farmed and wild salmon. We have so far no indications that PRV-infection leads to disease in wild salmon, this remains to be shown. But, as transmission of PRV is possible, it is not unlikely that other more virulent agents are transferred. If this occurs, and if it has an impact on wild fish, are important questions for future research.

## Supporting Information

Figure S1 **Phylogeny from initial maximum likelihood analysis of protein-coding PRV genome segment S2 in MEGA5.** Numbers above branches refer to bootstrap support based on 1000 random replicates. Samples are identified with ID-numbers, geographical origin, year of sampling and life-history. Colours are corresponding to geographical regions in Figure 3. Sequence from *Salmo trutta* is underlined. Groups I and III as defined in the phylogenetic analysis of S1 are indicated. Sample sequences not belonging to groups as defined by S1 are marked with an asterisk. Groups II and IV are not recovered in the same degree and therefore not indicated.
(TIF)

Figure S2 **Phylogeny from initial maximum likelihood analysis of protein-coding PRV genome segment S4 in MEGA5.** Numbers above branches refer to bootstrap support based on 1000 random replicates. Samples are identified with ID-numbers, geographical origin, year of sampling and life-history. Colours are corresponding to geographical regions in Figure 3. Sequence from *Salmo trutta* is underlined. Groups I, III and IV as defined by phylogenetic analysis of S1 are indicated. Sample sequences not belonging to groups as defined by S1 are marked with an asterisk. Group II is not recovered in the same degree and therefore not indicated.
(TIF)

Figure S3 **Maximum likelihood phylogeny of synonymous sites from the concatenated dataset.** The dataset contained protein-coding PRV genome segments S1, S2 and S4. Numbers above branches refer to Bayesian posterior probabilities and maximum likelihood bootstrap support, respectively. Samples are identified with ID-numbers, geographical origin and year of sampling. Sequences representing farmed Atlantic salmon are marked with their respective GenBank accessions. Colours are corresponding to geographical regions in Figure 3. Sequence from *Salmo trutta* is underlined.
(TIF)

Figure S4 **Maximum likelihood phylogeny of synonymous sites from PRV genome segment S1.** Numbers above branches refer to Bayesian posterior probabilities and maximum likelihood bootstrap support, respectively. Samples are identified with ID-numbers, geographical origin and year of sampling. Sequences representing farmed Atlantic salmon are marked with their respective GenBank accessions. Colours are corresponding to geographical regions in Figure 3. Sequence from *Salmo trutta* is underlined.
(TIF)
